# Epigenetic clocks in relapse after a first episode of schizophrenia

**DOI:** 10.1038/s41537-022-00268-2

**Published:** 2022-07-22

**Authors:** Àlex-González Segura, Llucia Prohens, Gisela Mezquida, Silvia Amoretti, Miquel Bioque, María Ribeiro, Xaquin Gurriarán-Bas, Lide Rementería, Daniel Berge, Roberto Rodriguez-Jimenez, Alexandra Roldán, Edith Pomarol-Clotet, Angela Ibáñez, Judith Usall, Maria Paz García-Portilla, Manuel J. Cuesta, Mara Parellada, Ana González-Pinto, Esther Berrocoso, Miquel Bernardo, Sergi Mas, 2EPS group, Jairo M. González-Díaz, Néstor Arbelo, Javier González-Peñas, Laura Pina-Camacho, Alba Diestre, Judit Selma, Iñaki Zorrilla, Purificación López, Amira Trabsa, Clara Monserrat, Luis Sanchez-Pastor, Aggie Nuñez-Doyle, Mar Fatjó-Vilas, Salvador Sarró, Anna Butjosa, Marta Pardo, Jose M. López-Ilundain, Ana M. Sánchez Torres, Jerónimo Saiz-Ruiz, Enriqueta Ochoa-Mangado, Olga RIevero, Concepción De-la-Cámara, Rafael Segarra Echevarría, Leticia González-Blanco

**Affiliations:** 1 Department of Clinical Foundations, Pharmacology Unit, University of Barcelona, Barcelona, Spain; 2 Institut d’investigacions Biomèdiques August Pi i Sunyer (IDIBAPs), Barcelona, Spain; 3 Barcelona Clínic Schizophrenia Unit (BCSU), Neuroscience Institute, Hospital Clínic de Barcelona, Barcelona, Spain; 4 Centro de Investigación Biomédica en red en salud Mental (CIBERSAM), Barcelona, Spain; 5 Department of Medicine, University of Barcelona, Barcelona, Spain; 6 Department of Psychiatry, Complejo Hospitalario de Navarra, Pamplona, Spain; 7 IdiSNA, Navarra Institute for Health Research, Pamplona, Spain; 8 Department of Child and Adolescent Psychiatry, Institute of Psychiatry and Mental Health, Hospital General Universitario Gregorio Marañón, IiSGM, School of Medicine, Universidad Complutense, Madrid, Spain; 9 Department of Psychiatry, Hospital Universitario de Alava, Vitoria, Spain; 10 Centro de Investigación Biomédica en Red de Salud Mental (CIBERSAM), Vitoria, Spain; 11 BIOARABA Health Research Institute, Vitoria, Spain; 12 Hospital del Mar Medicar Research Institute (IMIM), Barcelona, Spain; 13 Autonomous University of Barcelona, Barcelona, Spain; 14 Instituto de Investigación Sanitaria Hospital 12 de Octubre (imas12), Madrid, Spain; 15 CogPsy Group, Universidad Complutense de Madrid (UCM), Madrid, Spain; 16 Psychiatry Department, Institut d’Investigació Biomèdica-Sant Pau (IIB-SANT PAU), Hospital de la Santa Creu i Sant Pau, Barcelona, Spain; 17 FIDMAG Germanes Hospitalàries Research Foundation, Barcelona, Spain; 18 Department of Psychiatry, Hospital Universitario Ramón y Cajal, IRYCIS, Universidad de Alcalá, Madrid, Spain; 19 Parc Sanitari Sant Joan de Déu, Teaching, Research & Innovation Unit, Institut de Recerca Sant Joan de Déu, Barcelona, Spain; 20 Department of Psychiatry, Universidad de Oviedo, Oviedo, Principality of Asturias, Oviedo, Spain; 21 Instituto de Investigación Sanitaria del Principado de Asturias (ISPA), Oviedo, Principality of Asturias, Spain Instituto Universitario de Neurociencias del Principado de Asturias (INEUROPA), Oviedo, Principality of Asturias, Spain Servicio de Salud del Principado de Asturias (SESPA) Oviedo, Principality of Asturias, Oviedo, Spain; 22 Neuropsychopharmacology and Psychobiology Research Group, Department of Psychology, University of Cádiz, Cádiz, Spain; 23 Instituto de Investigación e Innovación Biomédica de Cádiz, INiBICA, Hospital Universitario Puerta del Mar, Cádiz, Spain; 24 Institut de Neuropsiquiatria, Parc de Salut Mar, Cádiz, Spain; 25 Department of Evolutionary Biology, Ecology and Environmental Sciences, Faculty of Biology, University of Barcelona, Barcelona, Spain; 26 Hospital Infanto-juvenil Sant Joan de Déu, Institut de Recerca Sant Joan de Déu, Esplugues de Llobregat, CIBERSAM, Barcelona, Spain; 27 Fundación Investigación Sanitaria INCLIVA; Departamento de Genética, Facultad de Biología, Universidad de Valencia, Burjassot, Valencia, Spain; 28 Hospital Clínico Universitario and Instituto de Investigación Sanitaria (IIS) Aragón, Zaragoza, Spain; 29 Department of Medicine and Psychiatry, Universidad de Zaragoza, Zaragoza, Spain; 30 Cruces University Hospital, BioCruces Health Research Institute, University of the Basque Country (UPV/EHU) Vizcaya, Vizcaya, Spain

**Keywords:** Epigenetics in the nervous system, Biomarkers, Schizophrenia

## Abstract

The main objective of the present study was to investigate the association between several epigenetic clocks, covering different aspects of aging, with schizophrenia relapse evaluated over a 3-year follow-up period in a cohort of ninety-one first-episode schizophrenia patients. Genome-wide DNA methylation was profiled and four epigenetic clocks, including epigenetic clocks of chronological age, mortality and telomere length were calculated. Patients that relapsed during the follow-up showed epigenetic acceleration of the telomere length clock (*p* = 0.030). Shorter telomere length was associated with cognitive performance (working memory, *r* = 0.31 *p* = 0.015; verbal fluency, *r* = 0.28 *p* = 0.028), but no direct effect of cognitive function or symptom severity on relapse was detected. The results of the present study suggest that epigenetic age acceleration could be involved in the clinical course of schizophrenia and could be a useful marker of relapse when measured in remission stages.

## Introduction

Schizophrenia has been associated with reduced life expectancy due to an increased suicide rate, poor health habits, and limited access to medical care, but especially due to chronic related pathologies^1–4^. Schizophrenia patients exhibit higher expression of age-related peripheral biomarkers of inflammation, oxidative stress and metabolic health^5^. The accelerated aging hypothesis of schizophrenia has been proposed to explain these observations, arguing that schizophrenia risk factors, either endogenous (i.e., genetic factors) or environmental (i.e., early life stressors), could accelerate the progressive biological changes of normal aging^5,6^.

According to recent research, aging can be defined in different ways and has several hallmarks that may play an integral role in the biological process of accelerated aging^7^. Several biomarkers of aging have been proposed in schizophrenia with contradictory outcomes, leading to the idea that schizophrenia may be associated with segmental aging, that is, some but not all features of aging may be accelerated^6^.

DNA methylation-based epigenetic clocks^8,9^ are validated predictors of chronological age across all tissues and across the entire lifespan. Increased or decreased epigenetic age relative to chronological age is termed as acceleration or deceleration. Age acceleration predicts an increased risk of age-related diseases^10^ and all-cause mortality^11^. Although aberrant DNA methylation has been consistently reported in schizophrenia^12^, no significant accelerated aging has been observed^13–18^. In fact, the largest study performed to date in whole blood and brain tissues identified a reduced age acceleration in patients who have schizophrenia, which contradicts the accelerated aging hypothesis of schizophrenia^19^. However, in recent years, numerous epigenetic clocks have been developed that appear to capture distinct aspects of aging, other than the classical prediction of chronological age, such as age-related morbidity and mortality^20^ or telomere length^21^. A recent large-scale DNA methylation study that simultaneously tested 14 epigenetic clocks covering different aspects of aging found that non-classical predictors of epigenetic aging (i.e., mortality telomere length clocks) were altered in schizophrenia cases^22^. Overall these results are consistent with the notion of segmental aging in schizophrenia. However, little is known about the effect that accelerated epigenetic age has on the clinical course of the disease.

The main objective of the present study was to investigate the association between several epigenetic clocks, covering different aspects of aging, with schizophrenia relapse evaluated over a 3-year follow-up period in a cohort of first-episode schizophrenia patients with less than 5 years of evolution. We hypothesized that relapse would be associated with accelerated aging, observable in clinical remission stages after a first episode of schizophrenia.

## Results

### Sample description

Table 1 shows the sociodemographic and clinical characteristics of the 91 participants at study entry, classified as non-relapse (those patients that had not experienced relapse after 3 years of enrollment) (*N* = 49) or relapse (those patients that relapsed during the 3-year follow-up) (*N* = 42). At study entry, relapsed patients were, on average, in the first year after the diagnosis of their first episode (0.90 ± 1.06), whereas non-relapsed patients had experienced their first episode one year and a half before (1.67 ± 1.44) (t89 = 2.82, *p* = 0.006).

**Table 1:** Demographic, clinical and cognitive data at baseline of the 91 participants in the study, classified as non-relapse (those patients that had not experienced a relapse after 3 years of enrollment) or relapse (those patients that relapsed during the 3-year follow-up).

	Non-Relapse	Relapse	Statistics
*N*	49	42	
Age (years), mean ± SD	26.6 ± 5.8	25.9 ± 5.9	t_89_ = 0.60 *p* = 0.545
Age at first diagnosis (years), mean ± SD	24.9 ± 5.6	24.7 ± 5.8	t_88_ = 0.19 *p* = 0.851
Time since first episode (years), mean ± SD	1.67 ± 1.44	0.90 ± 1.06	t_89_ = 2.82, *p* = 0.006
Gender, male, *N* (%)	38 (77.6)	27 (64.3)	X^2^_1_ = 1.95 *p* = 0.163
Ethnicity, Caucasian, *N* (%)	44 (89.8)	36 (85.7)	X^2^_1_ = 1.81 *p* = 0.771
Cannabis use, *N* (%)	5 (10.4)	8 (19.0)	X^2^_1_ = 1.35 *p* = 0.245
Tobacco use, *N* (%)	24 (49.0)	24 (57.1)	X^2^_1_ = 0.60 *p* = 0.437
Alcohol use, *N* (%)	18 (36.7)	21 (50.0)	X^2^_1_ = 1.65 *p* = 0.202
Antipsychotic CEDD, mean ± SD	254.8 ± 228.9	302.2 ± 305.8	t_89_ = −0.84 *p* = 0.401
Symptomatology			
Marder Positive symptoms, mean ± SD	11.6 ± 3.5	11.5 ± 3.9	t_89_ = 0.15 *p* = 0.882
Marder Negative symptoms, mean ± SD	14.2 ± 5.1	13.1 ± 5.6	t_89_ = 0.97 *p* = 0.333
Cognitive Domains^1^			
Working memory, mean ± SD	77.8 ± 15.1	73.9 ± 14.2	t_88_ = 1.24 *p* = 0.216
Verbal memory, mean ± SD	237.7 ± 68.9	221.4 ± 69.9	t_86_ = 1.07 *p* = 0.286
Executive function, mean ± SD	224.2 ± 34.5	228.5 ± 33.4	t_75_ = −0.47 *p* = 0.640
Visual memory, mean ± SD	88.4 ± 27.3	82.5 ± 23.7	t_87_ = 1.06 *p* = 0.289
Verbal fluency, mean ± SD	65.3 ± 15.2	62.0 ± 9.9	t_87_ = 1.18 *p* = 0.238
Sustained attention, mean ± SD	125.2 ± 22.6	135.3 ± 32.6	t_83_ = −1.67 *p* = 0.090
Processing speed, mean ± SD	68.1 ± 19.2	62.3 ± 17.8	t_88_ = 1.48 *p* = 0.142
Cognitive reserve, mean ± SD	61.4 ± 9.2	60.0 ± 7.6	t_83_ = 0.79 *p* = 0.430

CEDD, chlorpromazine equivalent daily dose.

^1^Identified through the principal components analysis.

### Epigenetic clocks in relapse

**Fig. 1:**
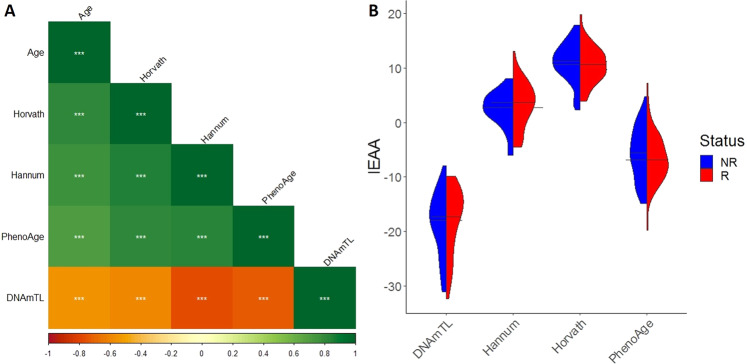
Epigentic age correlations and IEAA in relapse. **A** Correlation coefficient between the measured epigenetic clocks of chronological age (Horvath and Hannun clocks), mortality (PhenoAge) and telomere length (DNAmTL) and chronological age. (***p < 0.001). **B** Average IEAA of the measured epigenetic clocks of chronological age (Horvath and Hannun clocks), mortality (PhenoAge) and telomere length (DNAmTL) between patients that relapse (R) and those that do not relapse (NR).

In our cohort, the measured epigenetic clocks of chronological age (Horvath and Hannun clocks), mortality (PhenoAge) and telomere length (DNAmTL) were highly correlated with each other and with the chronological age (Fig. 1A). As might be expected, because telomere length decreases with age, a negative correlation between the DNAmTL clock and the other computed clocks and chronological age was observed.

Several factors may have an impact on DNA methylation, such as gender, socio-economic status, toxic habits (including tobacco, cannabis and alcohol), antipsychotic dose and time since first episode. In our cohort, none of these showed a significant association with the two measures of age acceleration in any epigenetic clock, except for the PhenoAge IEAA, which showed significant differences among genders (males −0.29 ± 4.62 vs. females 0.97 ± 5.13, t_89_ = 2.22 *p* = 0.029) (Supplementary Table 1).

**Fig. 2:**
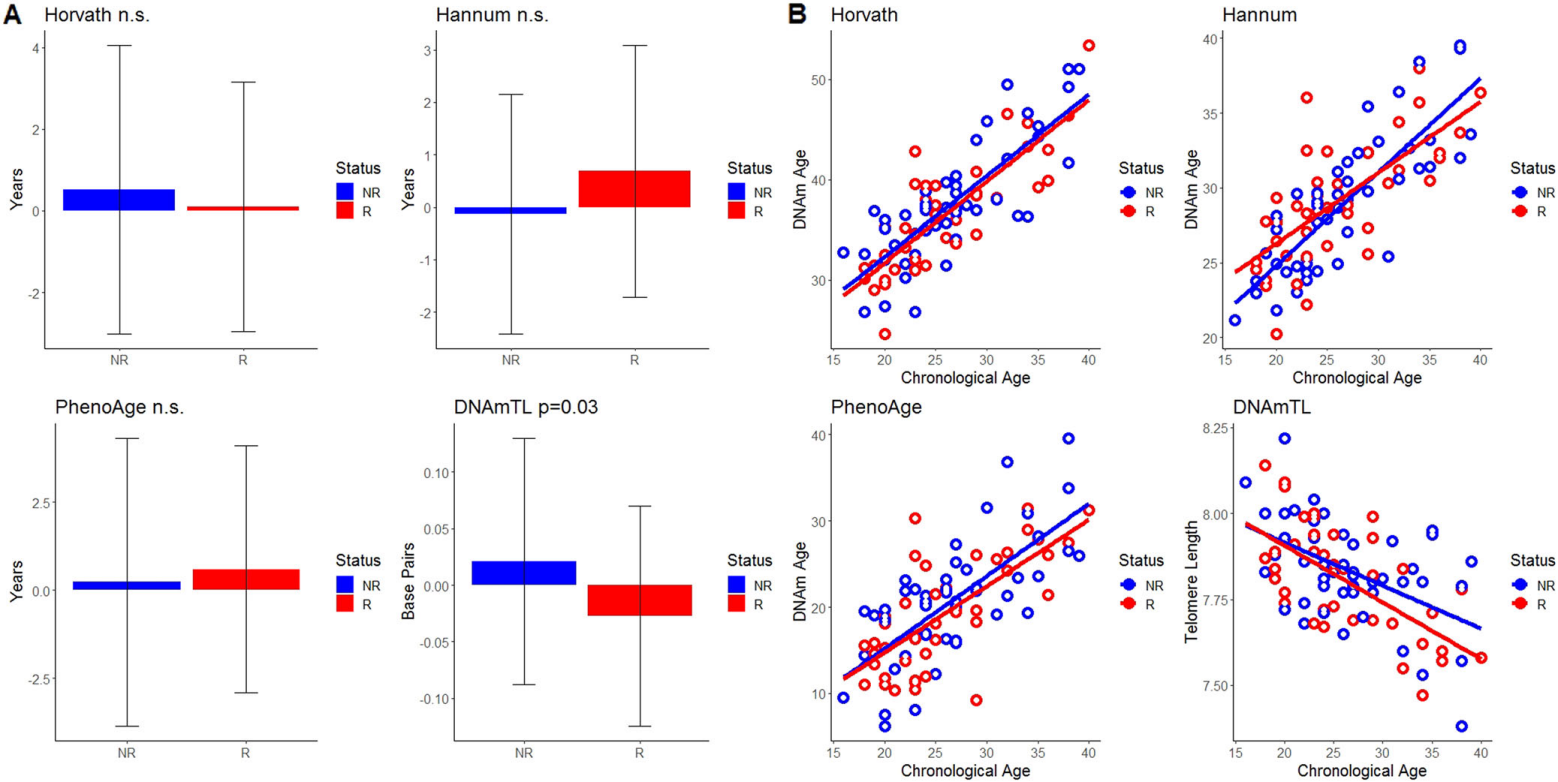
EEAA in relapse. Average EEAA of the epigenetic clocks (mean ± SD) (**A**) and chronological age compared to DNAm Age or telomere length (**B**) between patients that relapse (R) and those that do not relapse (NR).

We failed to find any acceleration or deceleration of the epigenetic clocks between relapsed and non-relapsed patients when we used the IEAA (Fig. 1B). However, when we adjusted for cell composition using the EEAA (Fig. 2), significant differences in acceleration of telomere length between non-relapsed patients (0.02 ± 0.11 base pairs) and relapsed patients (−0.03 ± 0.09 base pairs) were observed (t_89_ = 2.21, *p* = 0.030) (Fig. 2A). As mentioned previously, telomere length decreases with age, and for this reason we considered telomere length and DNAmTL to be accelerated in relapse (Fig. 2B). In order to explore the discrepancy between IEAA and EAA results we assessed the correlation between cell composition and epigenetic acceleration of the DNAmTL clock. IEAA showed significant positive correlation with CD4T (r = 0.40, *p* < 0.001), whereas as expected, EEAA showed no significant correlation with cell composition as it accounted for these differences.

### Epigenetic clocks and clinical data

As a secondary analysis we analyzed the partial correlations adjusting for gender, socio-economic status, toxic habits (tobacco, cannabis and alcohol), antipsychotic dose and time since first episode, has been performed to assess the correlations between the epigenetic age acceleration measures and symptom severity according to the standard Marder PANSS factors, the seven cognitive domains identified in the PCA analysis and the cognitive reserve. Only the EEAA of the DNAmTL clock showed significant correlations with working memory (*r* = 0.31 *p* = 0.015) and verbal fluency (*r* = 0.28 *p* = 0.028) (Supplementary Table 2).

Working memory and verbal fluency were considered for the mediation analysis, as they were significantly associated with the EEAA of the DNAmTL clock. However, mediation analyses did not provide evidence of a significant indirect effect of these mediator variables on relapse (Table 2).

**Table 2:** Results of the mediation analysis of selected clinical and cognitive variables in the association between EEAA DNAmTL and relapse.

Mediator	Direct Effect^1^ Estimate (95% CI) *p*-value	Total Effect^2^ Estimate (95% CI) *p*-value	Indirect Effect^3^ Estimate (95% CI) *p*-value
Working memory	−0.44 (−0.54, −0.24) 0.024	−0.44 (−0.54, −0.24) 0.020	−0.09 (−0.33, 0.26) 0.540
Verbal fluency	−0.45 (−0.55, −0.18) 0.040	−0.45 (−0.56, −0.26) 0.016	−0.08 (−0.34, 0.21) 0.604

^1^The effect of EEAA DNAmTL on relapse with the effect of the mediator.

^2^The effect of EEAA DNAmTL on relapse without the mediator.

^3^The total effect minus the direct effect.

## Discussion

To establish a relationship between epigenetic clocks and relapse after the remission of a first episode of psychosis we measured several epigenetic clocks to capture the complex and multifaceted process of aging^8,9,20,21^ in a cohort of patients with less than 5 years of evolution that were evaluated over a 3-year follow-up period. Patients that relapsed during the follow-up showed epigenetic acceleration of the telomere length clock, this is shorter telomeres than patients who had not relapsed. The significant results for the epigenetic estimator of telomere length, but not the other epigenetic clocks that predict chronological age or mortality, were consistent with the segmental aging hypothesis for schizophrenia, since not all features of accelerated aging are evident in these patients^6,22^.

The results of the present study suggest that epigenetic age acceleration could be involved in the clinical course of schizophrenia and could be a useful marker of relapse when measured in remission stages. This is consistent with a recent study that examined epigenetic clocks of chronological age and symptom severity in a schizophrenia sample^23^. The authors found a significant positive correlation between the EEAA measure and the SCL-90 psychotic score, with those patients with the worst scores having higher acceleration^23^. It is also consistent with previous studies that also showed associations between the EEAA and clinical symptoms and the course of the disease in other neuropsychiatric diseases such as posttraumatic stress disorder^24,25^.

Extrinsic epigenetic age acceleration measures capture both cell intrinsic methylation changes and extracelular changes in blood cell composition^26^. Therefore, by construction, reflects aspects of immunosenescence, as it captures age-related changes in blood cell composition, such as T lymphocyte populations, which underlie much of the age-related decline in the protective immune response^27^. Thus, the predictive significance of EEAA of the DNAmTL clock for relapse instead of IEAA probably reflects the fact that it assesses multiple aspects of the biological age of the immune system. Our results agreed with multiple evidences of dysregulation of the adaptive immunity cells in schizophrenia^28^. Moreover, a recent study identified a subtype of schizophrenia, using multi-omic measures, which exhibited widespread methylation level alterations among genes enriched in immune cell activity, as well as a higher proportion of neutrophils and lower proportion of lymphocytes^29^. This subtype had higher symptom severity and performed worse on cognitive measures, both factors that could be related to higher relapse rates.

Telomere length in leukocytes is one of the most studied hallmarks of aging in schizophrenia, having been used to demonstrate the accelerated aging hypothesis of this disease. Different studies have shown an association between telomere shortening and schizophrenia^30–39^, including a meta-analysis of case-control studies^40–42^. However, the evidence is inconclusive, with studies reporting no differences^43–50^ or even a longer telomere length in schizophrenia^51,52^.

Several studies have explored the role of telomere length in the course of the disease. Although different outcomes have been evaluated, our results are consistent with those that showed an association between shorter telomere length and poorer outcomes: no/poor response to antipsychotic medication^33,50,53^, no remission^35^, treatment-resistant schizophrenia^54^ and high symptom severity^43,48^.

Recently, telomere shortening has been associated with reduced hippocampal progenitor cell proliferation and the expression of genes regulating general cognitive functions^55^, reduced hippocampal volume^56^ and poor cognitive function in schizophrenia^39,43^. Cognitive reserve has been proposed as a mediator of symptomatology, functionality and cognition^57–60^, and having a high cognitive reserve predicts a better prognosis^61^. Based on the above, we hypothesized that, in schizophrenia, shorter telomeres reflect accelerated aging with reduced hippocampus proliferation that leads to worse cognitive function and poor response, which could translate into a higher risk of relapse after remission. Our results partially confirm this hypothesis, as shorter telomere length has been associated with poor cognition and relapse, but causality could not be demonstrated through mediation analysis. In our sample, no direct effect of cognitive function or symptom severity on relapse was detected. This could be related to the complexity of relapse as an outcome, being a complex phenotype that may not only be associated with the pathophysiological characteristics of patients but also with external factors (i.e., cannabis, lack of adherence). Moreover, the direct effect of shorter telomere on risk of relapse might not only be attributed to its effect on cognition but could also reflect the cumulative effect of environmental risk factors, such as psychosocial stress, obstetric complications or cannabis^62–66^, which in turn could also be related to relapse^67,68^.

These results should be interpreted in light of some limitations. Firstly, the sample size might limit the statistical power to detect a difference between groups or to stratify patients by antipsychotic medication. Secondly, although the Morinsky-Green scale was used to assess the adherence of the patients, our sample size was not large enough to stratify patients accordingly. Therefore, we were unable to separately analyze secondary relapse, which is commonly associated with non-adherence, and natural or primary relapse, which represents relapse in the absence of this influencer. Thirdly, our results regarding telomere length were obtained using an epigenetic predictor of length, not a direct measurement, so direct comparison with previous studies could be affected. Fourthly, due to the naturalistic design, drug treatment was not controlled, and the study participants maintained their usual treatment. Lastly, no specific scale was used to assess negative symptomatology, due to constraints associated with the PANSS scale. Although it is one of the most widely used measures of negative symptom severity, we acknowledge that it has several limitations. The use of Marder’s factors could solve some of these limitations. Finally, a limitation present in all CR studies undertaken on a psychiatric population is that as there is not yet a valid instrument to measure CR, criteria established and replicated in previous studies were followed. Besides these limitations, the strength of this study lies in the inclusion of a consistent well-characterized first-episode schizophrenia patient sample in remission due to its naturalistic and longitudinal design.

Our results provide preliminary evidence of the role of epigenetic aging in cognition and medium-term outcomes after remission. Further research is needed to validate our results in larger cohorts and to provide further explanation of the mechanistic relationship between epigenetic aging and relapse and the possible role of cognitive function and environmental variables.

## Material and methods

### Study design

This study is part of the project “Clinical and neurobiological determinants of second episodes of schizophrenia. Longitudinal study of first episode of psychosis” (PI11/00325) (2EPs Project), the aim of which is to identify and characterize the clinical, environmental and biological factors that predict a relapse. The 2EPs is a naturalistic, multicenter, coordinated and multimodal study of patients with a first psychotic episode of schizophrenia with less than 5 years of evolution and has a 3-year longitudinal-prospective follow-up design. The project includes six modules: general, neuroimaging, adherence, neurocognition, physical health and biological. Due to its main goals, the present study was framed within the general and biological modules. The first one aims to assess the presence or absence of relapses and includes clinical assessments. The aim of the biological module is to identify biomarkers potentially involved in second episodes (Bernardo et al., 2021; Gassó et al., 2021; Martínez-Pinteño et al., 2022; Rodríguez et al., 2022).

### Subjects

The inclusion criteria for the 2EPs Project were a) age 16–40 years at the time of first assessment (baseline visit); b) meeting diagnostic criteria according to DSM-IV-TR for schizophrenia or schizophreniform disorder (American Psychiatric Association, 1994); c) being in remission from the first psychotic episode (which should have occurred within the last 5 years), according to Andreasen’s criteria (Andreasen et al., 2005); d) not having relapsed after the first psychotic episode; e) speaking Spanish fluently, and f) signing the informed consent form. The exclusion criteria were a) having experienced a traumatic brain injury with loss of consciousness; b) presenting intellectual disability, with an intelligence quotient (IQ) < 70 and presenting malfunctioning and problems with adaptive processes, and/or c) presenting somatic pathology with mental repercussion.

Of the 223 patients recruited in the 2EPs Project, 119 participants (53.4%) completed the study. Ninety-one (76.5%) of these participated in the biological module and provided a biological sample for DNA methylation analysis at baseline.

The study was approved by the investigation ethics committees of all participating clinical centers. Informed consent was obtained from all participants. For children under the age of 18 years old, parents or legal guardians gave written informed consent before the study started, and patients assented to participate. When requested, participants in the study were given a report on the results of the tests. This study was conducted in accordance with the Declaration of Helsinki.

### Clinical assessment

At baseline demographic data and a complete personal and family history were collected in a systematic, self-devised interview. Diagnoses were determined according to the DSM-IV-TR criteria (American Psychiatric Association, 1994), using the SCID-I (Williams et al., 1992) or the Kiddie-SADS (Kaufman et al., 1997) depending on age.

Clinical symptomatology was assessed using the Spanish validated version of the Positive and Negative Syndrome Scale (PANSS) (Peralta & Cuesta, 1994). It has been argued that some PANSS items described as negative symptoms may be better described as cognitive deficits. Given this controversy, prior to our analyses we decided to use the Marder PANSS Factor Scores (Marder et al., 1997), which have different, more restrictive criteria for assessing both positive and negative symptomatology.

Pharmacological treatment was also recorded during all visits. The prescribed daily doses of antipsychotics were converted to chlorpromazine equivalent daily dose (CEED) following the method proposed by Leucht and colleagues (Leucht et al., 2016).

At baseline, a systematic survey of drug misuse habits was performed using part of the European adaptation of a multidimensional assessment instrument for drug and alcohol dependence: the multidimensional assessment tool European Addiction Severity Index (EuropAsi) (Kokkevi & Hartgers, 1995).

### Cognitive assessment

The neuropsychological battery, assessed at baseline, measured the following cognitive domains: (1) Estimated IQ from Block Design and Vocabulary of the Wechsler Adult Intelligence Scale (WAIS-III) (Wechsler, 1997); (2) Verbal learning and memory, evaluated using the California Verbal Learning Test (CVLT) (Delis et al., 1987); (3) Sustained attention, assessed using the Continuous Performance Test–II (CPT-II) (Conners et al., 2003), version 5, corrected by age and educational level; (4) Processing speed, assessed using the Trail Making Test (Form A) (TMT-A) (Reitan & Wolfson, 1995) and Digit Symbol (WAIS-III); (5) Executive functioning, evaluated using the Tower of London (TOL) (Shallice, 1982); (6) Working memory, based on the Digit Span Subtest and the Letter-Number Sequencing Subtest of the WAIS-III; (7) Verbal memory, assessed using the Brief Visuospatial Memory Test-Revised (Benedict & Groninger, 1995); (8) Verbal fluency, evaluated using semantic fluency (animals) (Peña-Casanova, 1991) and F-A-S tests (Loonstra et al., 2001); and (9) Emotional intelligence, evaluated using the Mayer-Salovey-Caruso Emotional Intelligence Test (MSCEIT) (Mayer et al., 2002).

A Principal Component Analysis (PCA) was performed between neuropsychological battery tests identifying the seven cognitive domains: verbal memory, visual memory, executive function, sustained attention, working memory, verbal fluency and processing speed (see Supplementary Table 3). Higher scores corresponded to better performance in all cognitive domains except for attention.

To assess cognitive reserve (CR) the three most commonly proposed proxy indicators of CR were used (Amoretti et al., 2016, 2018, 2022; de la Serna et al., 2013; González-Ortega et al., 2020): (1) The estimated premorbid IQ was calculated using the vocabulary subtest of the WAIS-III (Wechsler, 1997); (2) Education was assessed taking into account the degree of schooling attained and passed by the subject; and (3) Lifetime participation in leisure, social and physical activities was assessed using the Premorbid Adjustment Scale (PAS) (scholastic performance) and the Functioning Assessment Short Test (FAST) scale, which allowed us to assess specific life-domains such as interpersonal relationships and leisure time.

### Relapse definition

The main outcome variables were relapse rates. As inclusion criteria, patients fulfilled Andreasen’s criteria of symptomatic remission to enter the study, being considered at risk of relapse over the 3-year period (Andreasen et al., 2005).

Relapse was defined as when participants stop fulfilling these remission criteria for at least one week of the follow-up, scoring 4 or more in any of the 8 items of the PANSS Scale used to define these criteria: delusions, unusual thought content, hallucinatory behavior, mannerisms/posturing, blunted affect, social withdrawal, and lack of spontaneity. Hospitalization was also reported in every follow-up visit and considered a relapse when it was related to schizophrenia symptoms (and not to other causes). Follow-up visits to detect relapses were scheduled every 3 months, at which information was collected from the entire period between visits, and the patients, family members or caregivers and clinical teams in charge of the clinical follow-up could notify the research team of the possible relapse of a participant.

### Biological samples

Blood samples from each participant were collected at baseline in EDTA (BD Vacutainer K2EDTA tubes; Becton Dickinson, Franklin Lakes, New Jersey, USA). Genomic DNA was extracted using the MagNA Pure LC DNA Isolation Kit and a MagNA Pure LC 2.0 instrument (Roche Diagnostics GmbH, Mannheim, Germany) and DNA concentration and quality were measured using a NanoDrop 2000 spectrophotometer (Thermo Fisher Scientific, Surrey, CA). Genome-wide DNA methylation was profiled at the Centro Nacional de Genotipado (CEGEN-PRB3-ISCIII) using the Illumina Infinium MethylationEPIC BeadChip Kit.

### Methylation data collection

Raw intensity data (.IDAT) files were received and bioinformatics processes were conducted in house using the Chip Analysis Methylation Pipeline (ChAMP) Bioconductor package (Tian et al., 2017). Raw IDAT files were used to load the data into the R environment using the champ.load function, which also allows the probe quality control and removal steps to occur simultaneously. Probes with weak signals (*p* < 0.01) (*n* = 8441), cross-reactive probes (*n* = 11), non-CpG probes (*n* = 2914), probes with < 3 beads in at least 5% of the samples per probe (*n* = 7725), probes that bound to SNP (Single Nucleotide Polymorphisms) sites (*n* = 95,134), and sex chromosome probes (*n* = 16,445) were all considered problematic for the accurate detection of downstream methylation. After removing these probes, 735,248 probes remained for downstream analysis. β values were then normalized using the champ.norm function, specifically using the beta-mixture quartile method (BMIQ function). Next, the singular value decomposition (SVD) method was performed by champ.SVD to assess the amount and significance of the technical batch components in our dataset. Using the champ.runCombat function, combat algorithms were applied to correct for slide and array (significant components detected using the SVD method).

### Calculation of the epigenetic age with different clocks

Four epigenetic clocks, including epigenetic clocks of chronological age (Horvath and Hannun clocks), mortality (PhenoAge) and telomere length (DNAmTL) (details in Table 3), were calculated using the methylclock R package (Pelegi-Siso et al., 2021). Several authors demonstrated that the epigenetic clocks are resistant to the lack of CpG sites missing from the EPIC (McEwen et al., 2018). Briefly, the package extracts methylation levels of CpGs included in each clock from normalized and batched corrected methylation data. Subsequently, the coefficients obtained through elastic net in the prediction models of each of the clocks in the original papers were used to calculate DNA methylation age and epigenetic age acceleration in years, except for the DNAmTL clock, which measures telomere length in base pairs. For each clock we obtained the DNA methylation predicted age (DNAm age) in years, and two values of age acceleration: (1) the intrinsic epigenetic age acceleration (IEAA) (Chen et al., 2016), an age residual obtained after regressing chronological age on DNAm age, which captures cellular age acceleration independently of blood cell proportions that are known to change with age; and (2) the extrinsic epigenetic age acceleration (EEAA) (Chen et al., 2016), a set of residuals obtained after regressing chronological age and seven blood cell type proportions on DNAm age, which incorporates intrinsic measures as well as blood cell proportions. We estimated blood cell type proportion (CD4 T-lymphocytes, CD8 T-lymphocytes, monocytes, beta-cell, NK, neutrophils and eosinophils) using the reference panel from Reinius et al. (Reinius et al., 2012), as implemented in the meffil package (Min et al., 2018).

**Table 3:** Characteristics of the epigenetic clocks included in the present study.

Denotation	Reference	Phenotype	Number of CpG	Missing CpGs (%)^1^
Horvath	Horvath et al., 2013	Chronological age	353	30 (8.5)
Hannum	Hannum et al., 2013	Chronological age	71	12 (16.9)
PhenoAge	Levine et al., 2018	Mortality	513	16 (3.1)
DNAmTL	Lu et al., 2019	Telomere length	140	31 (22.1)

^1^Percentage of CpGs not included in the Illumina Infinium MethylationEPIC BeadChip Kit.

### Statistical analysis

Data were analyzed using SPSS 20.0 (statistical analysis software, IBM, Chicago, IL, USA). Two-tailed *p*-values < 0.05 were considered to be of statistical significance. Means and standard deviations were computed for continuous variables. The normality of continuous variables was tested using the Kolmogorov–Smirnov and Shapiro–Wilk tests, and the equality of the variance between groups was assessed using Levene’s test. The between-group difference in the continuous variables was analyzed using a Student’s *t*-test or Mann–Whitney U-test. The relationship between continuous variables was analyzed using partial correlations adjusted by potential confounding variables. A mediation analysis, using the Mediation R package, was carried out to evaluate the possible mediating role of clinical or neuropsychological variables on an association between epigenetic age acceleration and relapse (indirect effect). Regression coefficients were constructed using conventional mediation analysis models with a bootstrap sample size of 1000 and a 95% confidence interval.

## Supplementary information

Supplementary Data

## Data Availability

The clinical data that support the findings of this study are not openly available due to contain human data and are available from the corresponding author upon reasonable request.
