# Supplementary material for: Epigenetic clocks in relapse after a first episode of schizophrenia
Source: Schizophrenia (Heidelb). 2022 Jul 22;8(1):61. doi: 10.1038/s41537-022-00268-2 (PMC9307769; doi:10.1038/s41537-022-00268-2)
Supplement: Supplementary file 1 — Supplementary Data [file 41537_2022_268_MOESM1_ESM.docx]

**Supplementary** **Table 1.** Principal Component Analysis (PCA) for cognitive variables

| **Cognitive domain** | **Neuropsychological assessment** | **Pattern coefficients** | | | | | | | **Structure coefficients** | | | | | | | **Commu-nalities** |
| --- | --- | --- | --- | --- | --- | --- | --- | --- | --- | --- | --- | --- | --- | --- | --- | --- |
|  |  | **1** | **2** | **3** | **4** | **5** | **6** | **7** | **1** | **2** | **3** | **4** | **5** | **6** | **7** |  |
| **Verbal memory** | Long Delay Free Recall (CVLT) | **0.936** | -0.057 | 0.038 | -0.007 | 0.000 | -0.030 | 0.049 | **0.928** | 0.264 | 0.163 | -0.134 | 0.337 | 0.229 | 0.258 | 0.867 |
|  | Long Delay Cued Recall (CVLT) | **0.928** | -0.012 | 0.002 | 0.012 | 0.031 | -0.007 | 0.027 | **0.927** | 0.314 | 0.167 | -0.108 | 0.348 | 0.259 | 0.249 | 0.883 |
|  | Short Delay Free Recall (CVLT) | **0.926** | 0.028 | 0.035 | -0.006 | -0.024 | -0.084 | 0.024 | **0.915** | 0.324 | 0.156 | -0.131 | 0.309 | 0.174 | 0.237 | 0.846 |
|  | Short Delay Cued Recall (CVLT) | **0.915** | 0.004 | 0.038 | 0.023 | 0.006 | 0.002 | 0.025 | **0.939** | 0.306 | 0.134 | -0.119 | 0.369 | 0.255 | 0.255 | 0.862 |
|  | Total A5 (CVLT) | **0.847** | 0.014 | -0.018 | -0.018 | 0.030 | 0.044 | -0.053 | **0.861** | 0.290 | 0.104 | -0.132 | 0.330 | 0.273 | 0.175 | 0.746 |
|  | Total Immediate Recall (CVLT) | **0.842** | 0.056 | -0.061 | -0.020 | -0.004 | 0.119 | -0.059 | **0.872** | 0.327 | 0.067 | -0.141 | 0.317 | 0.340 | 0.180 | 0.78 |
| **Visual memory** | Total Delay Recall (BVMT-R) | 0.115 | **0.920** | -0.079 | 0.024 | -0.024 | -0.022 | 0.066 | 0.403 | **0.957** | 0.023 | -0.101 | 0.263 | 0.128 | 0.336 | 0.935 |
|  | Total Immediate Recall (BVMT-R) | -0.110 | **0.806** | 0.110 | -0.071 | 0.054 | 0.153 | 0.036 | 0.249 | **0.833** | 0.196 | -0.171 | 0.289 | 0.266 | 0.304 | 0.745 |
| **Executive function** | Total move (TOL) | 0.004 | -0.032 | **0.911** | -0.003 | 0.022 | 0.012 | 0.009 | 0.133 | 0.066 | **0.913** | -0.021 | 0.127 | 0.102 | 0.076 | 0.835 |
|  | Total correct (TOL) | 0.040 | -0.199 | **0.851** | -0.028 | -0.103 | 0.052 | 0.114 | 0.101 | -0.089 | **0.839** | -0.044 | 0.010 | 0.116 | 0.108 | 0.754 |
|  | Execution time (TOL) | 0.025 | 0.357 | **0.684** | 0.039 | 0.101 | -0.077 | -0.128 | 0.216 | 0.405 | **0.716** | 0.002 | 0.221 | 0.040 | 0.042 | 0.654 |
| **Sustained attention** | Perseverations (CPT-II) | -0.036 | 0.040 | 0.007 | **0.803** | 0.060 | -0.255 | 0.121 | -0.149 | -0.045 | -0.018 | **0.803** | -0.010 | -0.289 | -0.024 | 0.718 |
|  | Variability (CPT-II) | 0.003 | 0.000 | -0.085 | **0.801** | -0.158 | -0.092 | -0.087 | -0.221 | -0.176 | -0.134 | **0.833** | -0.265 | -0.218 | -0.283 | 0.764 |
|  | Hit RT (CPT-II) | -0.018 | -0.071 | 0.070 | **0.747** | 0.033 | 0.375 | -0.073 | -0.038 | -0.117 | 0.080 | **0.734** | 0.034 | 0.300 | -0.130 | 0.686 |
| **Working memory** | Digits (WAIS-III) | -0.041 | 0.009 | 0.016 | -0.059 | **0.866** | -0.003 | 0.056 | 0.298 | 0.245 | 0.116 | -0.116 | **0.877** | 0.206 | 0.341 | 0.778 |
|  | Letter-Number Sequencing (WAIS-III) | 0.071 | -0.045 | -0.043 | 0.030 | **0.850** | -0.012 | 0.000 | 0.349 | 0.190 | 0.059 | -0.025 | **0.854** | 0.193 | 0.271 | 0.736 |
| **Verbal fluency** | Categorical (Animal naming) | 0.091 | 0.173 | 0.018 | -0.140 | -0.046 | **0.687** | 0.189 | 0.385 | 0.354 | 0.123 | -0.256 | 0.264 | **0.771** | 0.390 | 0.726 |
|  | Phonemic (F‐A‐S) | 0.182 | 0.046 | 0.038 | -0.003 | 0.218 | **0.615** | -0.004 | 0.447 | 0.247 | 0.151 | -0.097 | 0.442 | **0.724** | 0.236 | 0.639 |
| **Processing speed** | TMT-A | 0.035 | 0.074 | 0.068 | -0.038 | 0.035 | -0.118 | **0.869** | 0.265 | 0.338 | 0.135 | -0.178 | 0.333 | 0.073 | **0.900** | 0.835 |
|  | Digit Symbol (WAIS-III) | 0.013 | 0.054 | -0.047 | 0.056 | 0.099 | 0.283 | **0.662** | 0.290 | 0.301 | 0.045 | -0.081 | 0.391 | 0.425 | **0.751** | 0.670 |

Abbreviations: CVLT=California Verbal Learning Test; BVMT-R= Brief visuospatial memory test-revised; TOL= Tower of London; CPT-II= Continuous Performance Test–II; RT=Reaction Time; WAIS-III= Wechsler Adult Intelligence Scale; TMT-A= Trail making test (Form A).

The 20 neuropsychological variables were subjected to principal components analysis (PCA) using SPSS version 25. The Kaiser-Meyer-Olkin value was 0.776, exceeding the recommended value of 0.6 and Bartlett’s Test of Sphericity reached statistical significance, supporting the factorability of the correlation matrix. Principal components analysis revealed the presence of seven components with eigenvalues exceeding 1, explaining 34.11%, 10.73%, 9.55%, 8.51%, 6.90%, 4.40% and 3.88% of the variance respectively. The rotated solution revealed the presence of simple structure with seven components showing a number of strong loadings.

**Supplementary** **Table 2.** Summary statistics of the impact on age acceleration of potential confounding variables (gender, socio-economic status, toxic habits (including tobacco, cannabis and alcohol) and antipsychotic dose).

|  | **Horvath** | | **Hannum** | | **PhenoAge** | | **DNAmTL** | |
| --- | --- | --- | --- | --- | --- | --- | --- | --- |
|  | **IEAA** | **EEAA** | **IEAA** | **EEAA** | **IEAA** | **EEAA** | **IEAA** | **EEAA** |
| **Gender (male/female)^1^** | t_89_=-0.40 p=0.687 | t_89_=-0.52 p=0.603 | t_89_=-0.15 p=0.880 | t_89_=1.53 p=0.130 | t_89_=1.14 p=0.254 | **t_89_=2.22 p=0.029** | t_89_=1.45 p=0.150 | t_89_=-0.26 p=0.794 |
| **Socio-economic Status^2^** | F_4_=0.26 p=0.897 | F_4_=0.26 p=0.901 | F_4_=0.65 p=0.624 | F_4_=0.43 p=0.782 | F_4_=0.30 p=0.872 | F_4_=0.43 p=0.784 | F_4_=0.83 p=0.505 | F_4_=1.13 p=0.344 |
| **Cannabis use (yes/no) ^1^** | t_89_=0.64 p=0.521 | t_89_=0.87 p=0.383 | t_89_=-0.57 p=0.564 | t_89_=0.14 p=0.886 | t_89_=-0.42 p=0.676 | t_89_=0.41 p=0.680 | t_89_=1.48 p=0.142 | t_89_=0.77 p=0.439 |
| **Tobacco use (yes/no) ^1^** | t_89_=1.55 p=0.125 | t_89_=1.60 p=0.112 | t_89_=1.20 p=0.231 | t_89_=1.09 p=0.275 | t_89_=0.86 p=0.391 | t_89_=1.10 p=0.273 | t_89_=0.47 p=0.640 | t_89_=0.94 p=0.348 |
| **Alcohol use (yes/no) ^1^** | t_89_=-0.14 p=0.887 | t_89_=-0.06 p=0.952 | t_89_=-0.11 p=0.908 | t_89_=-0.02 p=0.985 | t_89_=0.07 p=0.945 | t_89_=0.74 p=0.459 | t_89_=0.81 p=0.420 | t_89_=0.10 p=0.920 |
| **Antipsychotic CEDD^3^** | r=-0.06 p=0.561 | r=-0.07 p=0.507 | r=-0.00 p=0.959 | r=-0.09 p=0.361 | r=0.07 p=0.474 | r=0.00 p=0.951 | r=0.00 p=0.561 | r=0.04 p=0.691 |
| **Time since first episode (years)** | r=-0.06 p=0.561 | r=-0.07 p=0.507 | r=-0.00 p=0.959 | r=-0.09 p=0.361 | r=0.07 p=0.474 | r=0.00 p=0.951 | r=0.00 p=0.561 | r=0.04 p=0.691 |

**^1^**Student’s t-test; **^2^**ANOVA test; **^3^**Pearson’s correlation coefficient

**Supplementary Table 3.** Correlation between the epigenetic age acceleration measures and symptom severity according to the standard Marder PANSS factors, the seven cognitive domains identified in the PCA analysis and the cognitive reserve.

|  | | **Horvath** | | **Hannum** | | **PhenoAge** | | **DNAmTL** | |
| --- | --- | --- | --- | --- | --- | --- | --- | --- | --- |
|  | | **IEAA** | **EEAA** | **IEAA** | **EEAA** | **IEAA** | **EEAA** | **IEAA** | **EEAA** |
| **Cognitive Reserve** | r | -.204 | -.155 | -.229 | -.231 | -.161 | -.213 | .175 | .173 |
|  | p-value | .114 | .234 | .075 | .073 | .214 | .099 | .177 | .182 |
| **Marder Positive Symptoms** | r | -.042 | -.080 | -.099 | -.086 | -.022 | -.015 | .051 | .012 |
|  | p-value | .749 | .542 | .446 | .508 | .864 | .911 | .695 | .926 |
| **Marder Negative Symptoms** | r | -.003 | -.052 | .048 | .067 | .029 | -.030 | -.130 | -.189 |
|  | p-value | .981 | .692 | .714 | .611 | .823 | .818 | .316 | .145 |
| **Working Memory** | r | -.242 | -.246 | -.241 | -.241 | -.143 | -.218 | .222 | **.310** |
|  | p-value | .061 | .056 | .061 | .062 | .271 | .091 | .074 | **.015** |
| **Verbal Memory** | r | -.028 | .010 | -.040 | -.048 | -.071 | -.042 | .167 | .182 |
|  | p-value | .832 | .941 | .758 | .711 | .584 | .746 | .199 | .161 |
| **Executive Function** | r | -.173 | -.201 | -.002 | -.019 | .012 | -.013 | -.119 | -.053 |
|  | p-value | .183 | .120 | .989 | .882 | .926 | .922 | .359 | .683 |
| **Visual Memory** | r | -.235 | -.251 | -.142 | -.198 | -.004 | -.123 | .095 | .163 |
|  | p-value | .068 | .051 | .274 | .126 | .975 | .344 | .468 | .210 |
| **Verbal Fluency** | r | -.100 | -.093 | -.151 | -.175 | -.113 | -.142 | .220 | **.282** |
|  | p-value | .442 | .476 | .247 | .177 | .385 | .276 | .089 | **.028** |
| **Sustained attention** | r | .147 | .131 | .143 | .114 | .131 | .023 | -.159 | -.158 |
|  | p-value | .259 | .313 | .273 | .383 | .315 | .858 | .222 | .224 |
| **Processing speed** | r | -.249 | -.232 | -.235 | -.244 | -.204 | -.237 | .198 | .183 |
|  | p-value | .056 | .079 | .066 | .059 | .114 | .065 | .126 | .158 |
